# An interprofessional core elective module on the scholarly presentation of projects: implementation in an online format

**DOI:** 10.3205/zma001486

**Published:** 2021-06-15

**Authors:** Franziska Krakau, Lea Doll, Anika Mitzkat

**Affiliations:** 1 Universitätsklinikum Heidelberg, Abteilung Allgemeinmedizin & Versorgungsforschung, Heidelberg, Germany

**Keywords:** interprofessional education, blended learning, asynchronous teaching formats, synchronous teaching formats, academic competencies

## Abstract

**Background:** In the bachelor degree program Interprofessional Health Care that combines professional training and study, students work part-time in their chosen professions after completing training. The increase in students’ working hours due to COVID-19 and the switch to a digital teaching format raised the question as to how a successful and flexible educational concept can be created online in this context.

A blended-learning strategy in combination with a competency model for interprofessional learning was chosen as theoretical reference point for implementation. Based on a module for academic poster presentation in front of an interprofessional plenum, the sequence of the learning process organization in the phases “kick-off”, “self-directed learning” and “online seminar” is exemplified and discussed with regard to its suitability for digital interprofessional teaching.

**Implementation: **During implementation it was important to clearly define the module’s scope and sequence at the very beginning. The use of screencasts enabled students to individually pace their learning during the preparatory self-directed learning phase. Embedding assignments in the screencasts served to aid students in their learning. The synchronous exchange in interprofessional small groups was experienced as profitable for the own poster production. Several students perceive their own poster presentation in digital format as an increase in competence and a basis for future academic presentations.

**Summary:** In summary, the entire interprofessional module was successfully implemented digitally in the phases “kick-off”, “self-directed learning” and “online seminar”. For synchronous learning, virtual small group workspaces seem particularly suitable for learner activation. The practical implementation of the acquired competencies in the form of the poster presentation is crucial for ensuring the learning success.

## Zusammenfassung

**Hintergrund:** Im ausbildungsintegrierenden Bachelorstudiengang „Interprofessionelle Gesundheitsversorgung“ arbeiten Student_innen nach Ausbildungsende in Teilzeit in ihrem erlernten Gesundheitsberuf. Die durch COVID-19 bedingte Aufstockung der Arbeitszeit der Student_innen und die Lehrformatsumstellung auf digitale Formate warf die Fragen auf, wie ein gelingendes und flexibles Lehr-Lernkonzept in diesem Kontext online gestaltet werden kann.

Für die Umsetzung wurden als theoretische Bezugspunkte ein Blended Learning Konzept in Kombination mit einem Kompetenzmodell interprofessionellen Lernens gewählt. Anhand eines Moduls zur wissenschaftlichen Posterpräsentation vor einem interprofessionellen Plenum wird der Ablauf der Lernprozessorganisation in den Phasen „Kick-off“, „Selbstgesteuertes Lernen“ und „Online-Seminar“ exemplarisch dargestellt und hinsichtlich der Eignung für die digitale interprofessionelle Lehre diskutiert.

**Umsetzung: **Bei der Umsetzung war die Klärung des Ablaufs und eines verbindlichen Rahmens zu Beginn des Moduls wichtig. Mittels Screencasts konnte danach eine individuelle Festlegung des Lerntempos in der selbstgesteuerten vorbereitenden Lernphase stattfinden. Die Einbettung von Arbeitsaufträgen in die aufgezeichneten Screencasts unterstütze die Student_innen beim Lernen. Der synchrone Austausch in interprofessionellen Kleingruppen wurde als gewinnbringend für die eigene Postererstellung erlebt. Einige Student_innen empfanden die eigene Posterpräsentation in digitalem Format als Kompetenzzuwachs und Basis für spätere wissenschaftliche Präsentationen.

**Fazit: **Zusammenfassend zeigte sich, dass das gesamte interprofessionelle Modul digital in den Phasen „Kick-off“, „Selbstgesteuertes Lernen“ und „Online-Seminar“ erfolgreich umsetzbar war. Für das synchrone in Lernen erscheinen insbesondere virtuelle Kleingruppenarbeitsräume zur Lerneraktivierung geeignet. Die praktische Umsetzung der erworbenen Kompetenzen in Form der Posterpräsentation ist entscheidend für die Absicherung des Lernerfolges.

## Background

In the bachelor degree program Interprofessional Health Care (IPG) [1], members of nine different health care professions learn from, with and about each other [2]. After completing the simultaneous, yet separate training, students begin working part-time as young professionals in their respective occupations in clinical health care. At the beginning of the COVID-19 pandemic many students had to increase their work hours up to 100% due to shortfalls in personnel, which increased the double burden of work and study. Teaching in the degree program was completely switched to online formats.

The core elective project module involves 135 hours of cooperation on a research project, a project promoting quality, or a project on international exchange. The module’s aim is to have students gather profession-specific experience in project work and, at the same time, acquire both academic and interprofessional competencies. For this purpose, a seminar (15 hours in total) is held alongside the project, in which the students are taught academic competencies for the submission, presentation and discussion of conference contributions and in which the interprofessional exchange about the project work is stimulated. Learning is assessed by preparation and presenting an academic poster in front of an interprofessional group.

The following questions arose from this initial situation:

1. How can the core elective project module be designed to be as flexible as possible for students in order to keep the double burden of work and study as low as possible?
2. Which online formats can be used to exemplarily implement a complete online teaching-learning concept for the teaching of academic competencies in relation to the presentation of the results of a project?

**Figure 1:**
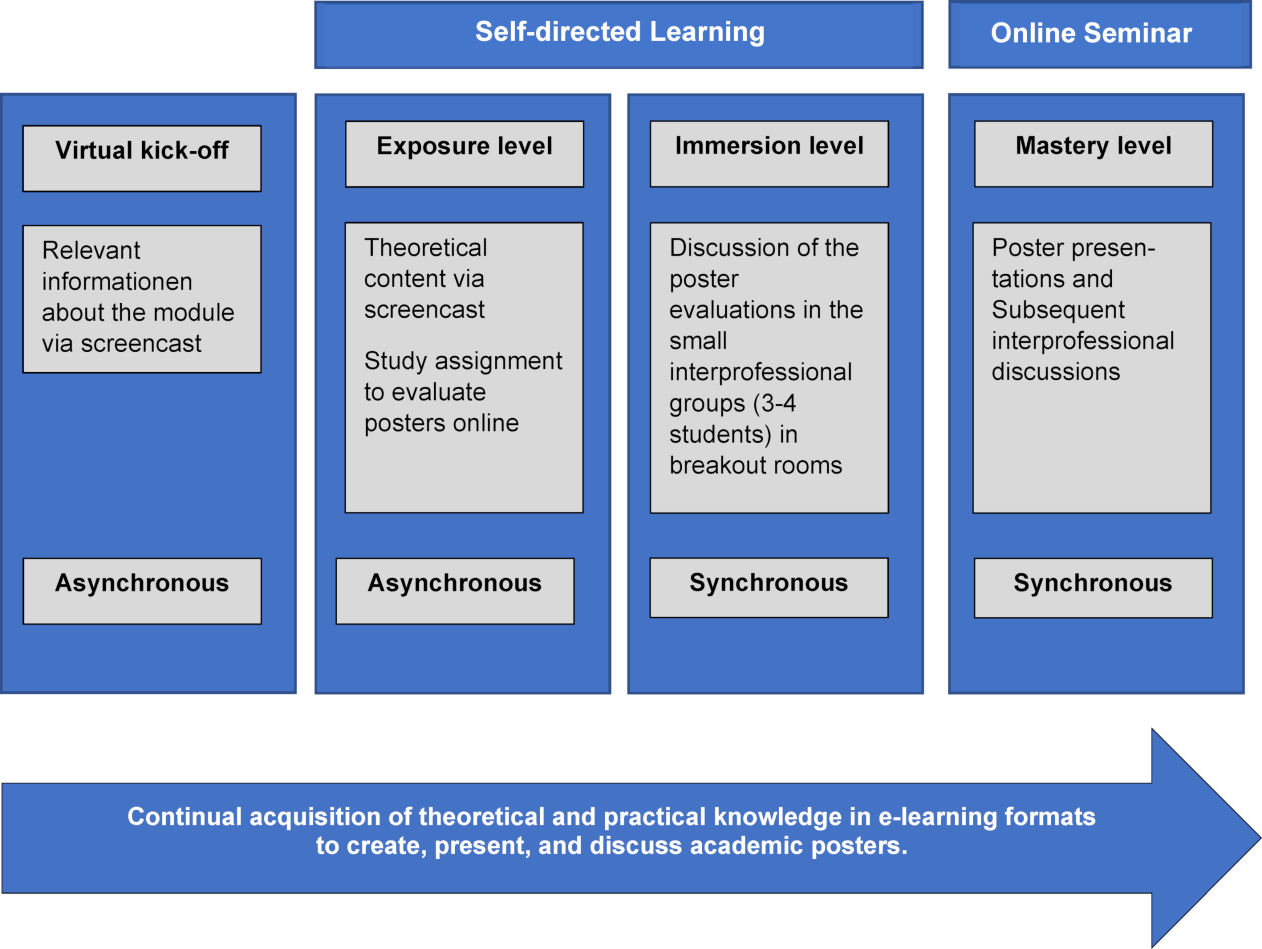
Organization of the learning process for a virtual blended-learning concept (based on Erpenbeck et al. [3] and Charles et al. [5])

Two theoretical reference points formed the basis for implementing the online teaching: a blended learning concept consisting of alternating synchronous and asynchronous online formats [3] and a competency model for interprofessional learning [4], [5]. This competency model assumes that interprofessional learning takes place in three successive phases depending on the educational level of the students (see figure 1 (Fig. 1)).

The first phase entails the asynchronous teaching of knowledge-based content (exposure level). In the second, synchronous phase, the focus is on activating the students to apply their acquired knowledge and to engage in discussions (immersion level). Both phases can be categorized as self-directed learning within the concept of blended learning. The third, also synchronous, phase targets interprofessional interaction between students and was implemented as an online seminar in the blended-learning process (mastery level). For example, a student with a background in medical laboratory science (MTLA) would present the results of their collaboration on a research project in the laboratory, while a student with expertise in physiotherapy would present a project on quality assurance in ambulatory care.

Notwithstanding how the module is realized interprofessionally in the IPG degree program, this strategy to impart scholarly competencies is also transferrable to monoprofessional groups.

The implementation of the teaching concept is illustrated in the following.

## Didactic-methodical implementation of the online interprofessional elective module

For the online teaching in the 2020 summer term, the blended learning process was structured based on Erpenbeck, Sauter and Sauter [3], and with special regard for the opportunities offered by interprofessional education [5] (see figure 1 (Fig. 1)). For the entire module, the e-learning platform moodle and heiCONF for synchronous video conferencing were used. For the asynchronous knowledge transfer, lecture slides were enhanced with audio narration (screencast) and paired with study assignments to prepare students for the subsequent synchronous learning phase.

Twenty-one students took the module during the 2020 summer term.

### Virtual kick-off

At the beginning of the term the students received all of the relevant information about the module’s structure and organization via screencast. Particular attention was paid to the study assignments and the organizational and technical details of the online course. Individual appointments were held with students by telephone or video conference to clarify questions about their projects.

#### Self-directed learning

To get started with self-guided learning, screencasts were made available so that students could work toward and fulfill the knowledge-based learning objectives. Among these were the requirements regarding the form and content for poster presentations at scientific conferences, the submission procedure, and information on the “do’s and don’ts” when presenting a poster. A follow-up assignment was given at the end of the asynchronous phase. The activity-based study assignment had students evaluate three academic posters according to a defined set of criteria and justify their evaluations. The evaluation criteria corresponded to those applied to learning assessment and were made available to the students. The posters were accessible via moodle.

The results were then compiled during a video conference. The study assignment was taken up as a topic and the students were brought together in small interprofessional groups of 3-4 students in virtual group spaces (“breakout rooms”). The interaction between the students was encouraged through clear instructions for sharing thoughts and information about the evaluated posters. One person in each group was selected to take notes and another to serve as the group‘s spokesperson who later presented the results in the plenary session. In addition to exchanging cross-professional scientific aspects, the focus was on linking different professional perspectives and identifying overlaps in order to work through and complete a shared task. The instructors were available via chat to answer questions and were able to enter and participate in the separate breakout rooms as needed (questions of the students, halting of the exchange in break-out sessions).

The spokespersons from the small groups then presented the results of their group’s virtual poster evaluations and ensuing discussion in the plenum. As this was done, suggestions for improvement were worked on together to be applied later to the students’ own posters. The session information was recorded by writing notes down on a virtual whiteboard.

The self-directed learning phase can be described on two competency levels: On the basic level (exposure), the emphasis was on the student’s own role and responsibility [5]. The students were required to assess their own level of knowledge and justify the evaluation of an academic poster based on a set of criteria. The assignment was given with a set of concrete instructions and defined as a pre-requisite for the subsequent learning steps. Working through the screencasts and the study assignment was then done individually by the students on a flexible basis.

At the intermediate level (immersion), the focus was turned to activating the students [5]. They were asked to explain and discuss the results with their peers and to reach a consensus on the final evaluation. To accomplish this, cooperative learning activities were encouraged as part of using the breakout rooms. A clear assignment along with instructions on presenting the results ensured that the students’ interactions were goal-oriented under the guidance of a spokesperson.

#### Online seminar

Three online seminar sessions were held over the remaining course of the term in which the students presented their own projects in the form of an e-poster that served to assess student learning. Addressing questions in the subsequent interprofessional discussion was part of this assessment.

In the context of interprofessional learning, with a focus on the interaction between students in this phase, previously acquired knowledge was applied by the students when virtually presenting their own posters, leading to the achievement of a practice level (mastery) [5].

## Lessons learned/conclusion

Student feedback was gathered in the online seminar chat, as flash feedback via survey, and individually per email or telephone within the scope of general advising.

The students especially liked having the option to determine their own pace when working through the screencasts. The instructors also observed that the screencasts and the study assignments were very dependably completed by the students and that all of the students showed up well prepared for the synchronous online units. The student discussions and presentations in the small interprofessional groups were experienced as a beneficial learning format, and putting the session notes on the whiteboard was found to be a helpful visualization of the learned material.

The majority of students, however, made the critical comment that they wished to present their posters in person in the classroom setting. The instructors also observed that the discussions of the presentations given by students for the learning assessment were more halting than is the case in a classroom.

The following conclusions can be drawn from this:

- The concept of blended learning can also be implemented in interprofessional groups in an exclusively online course.
- Flexibility between work and study is facilitated by asynchronous, self-directed learning units.
- Setting a defined scope at the beginning of the module and having a means to assess learning by using assignments and feedback are important for the self-directed learning phase.
- Encouraging student activity and interaction is critical to learning. Assignments that are completed together in the breakout rooms are particularly suitable for accomplishing this.
- The practical application of theoretical knowledge represents the highest level of the learning process and is vital to acquiring practical competencies.

A structured prospective evaluation of the module is planned following its piloting described above. An online survey covering not only the acquisition of professional competencies, but also interprofessional learning will be conducted in the 2020/21 winter term. If the pandemic situation allows, the poster presentations at the end of the module will be held in person.

## Competing interests

The authors declare that they have no competing interests.
